# Identification of late assembly domains of the human endogenous retrovirus-K(HML-2)

**DOI:** 10.1186/1742-4690-10-140

**Published:** 2013-11-19

**Authors:** Claudia Chudak, Nadine Beimforde, Maja George, Anja Zimmermann, Veronika Lausch, Kirsten Hanke, Norbert Bannert

**Affiliations:** 1 Department for HIV and other Retroviruses, Robert Koch Institute, Nordufer 20, 13353 Berlin, Germany; 2 Robert Koch Institute, Federal Information Centre for Biological Security, DGZ Ring 1, 13086 Berlin, Germany

**Keywords:** HERV-K(HML-2), Gag protein, Late assembly domain, Budding, Retrovirus, Alix, Tsg101, Endogenous retrovirus

## Abstract

**Background:**

Late assembly (L)-domains are protein interaction motifs, whose dysfunction causes characteristic budding defects in enveloped viruses. Three different amino acid motifs, namely PT/SAP, PPXY and YPX_n_L have been shown to play a major role in the release of exogenous retroviruses. Although the L-domains of exogenous retroviruses have been studied comprehensively, little is known about these motifs in endogenous human retroviruses.

**Results:**

Using a molecular clone of the human endogenous retrovirus K113 that had been engineered to reverse the presumed non-synonymous postinsertional mutations in the major genes, we identified three functional L-domains of the virus, all located in the Gag p15 protein. A consensus PTAP tetrapeptide serves as the core of a main L-domain for the virus and its inactivation reduces virus release in HEK 293T cells by over 80%. Electron microscopy of cells expressing the PTAP mutant revealed predominantly late budding structures and budding chains at the plasma membrane. The fact that this motif determines subcellular colocalization with Tsg101, an ESCRT-I complex protein known to bind to the core tetrapeptide, supports its role as an L-domain. Moreover, two YPX_n_L motifs providing additional L-domain function were identified in the p15 protein. One is adjacent to the PTAP sequence and the other is in the p15 N-terminus. Mutations in either motif diminishes virus release and induces an L-domain phenotype while inactivation of all three L-domains results in a complete loss of particle release in HEK 293T cells. The flexibility of the virus in the use of L-domains for gaining access to the ESCRT machinery is demonstrated by overexpression of Tsg101 which rescues the release of the YPX_n_L mutants. Similarly, overexpression of Alix not only enhances release of the PTAP mutant by a factor of four but also the release of a triple mutant, indicating that additional cryptic YPX_n_L domains with a low affinity for Alix may be present. No L-domain activity is provided by the proline-rich peptides at the Gag C-terminus.

**Conclusions:**

Our data demonstrate that HERV-K(HML-2) release is predominantly mediated through a consensus PTAP motif and two auxiliary YPX_n_L motifs in the p15 protein of the Gag precursor.

## Background

The Gag precursor plays an essential role in retroviral assembly and release [1,2]. It contains domains that mediate an association with the lipid bilayer, Gag-Gag subunit-interactions and egress from the producer cell. Successful budding ending with membrane scission to release retroviral particles depends on short peptide motifs of this protein termed ‘late (L)-domains’. Deletions or explicit mutations in viral L-domains freeze the budding step and prevent the separation of virions from the plasma membrane [3,4].

To date, three characteristic classes of L-domains have been defined, namely PT/SAP, YPX_n_L and PPXY. An L-domain extends beyond the largely preserved core amino acids. A functional PT/SAP class of L-domains can be as large as 12 amino acids. L-domains function by directly or indirectly linking the Gag precursor proteins to the cellular ESCRT machinery principally involved in the endosomal sorting of cargo proteins and the biogenesis of multivesicular bodies. This machinery consists of about 25 cellular proteins that form four major complexes termed ESCRT-0, -I, -II and -III [5].

The three classes of L-domains found in viruses interact with different sorting complex proteins to gain access to the ESCRT pathway. The PT/SAP motif interacts with Tsg101 (tumour susceptibility gene 101), an ESCRT-I complex component, which was identified in yeast two-hybrid experiments as an HIV-1 p6-interacting protein. The depletion of Tsg101 by small interfering RNA interrupts HIV-1 budding to a large extent [6]. On the other hand, the PPXY motif functions through a direct recruitment of Nedd4 (neuronal precursor cell-expressed developmentally down-regulated-4)-like ubiquitin ligases by binding to its multiple WW domains [7]. Ubiquitinylation is a critical step in processes involving the ESCRT pathway and viral budding [8-10]. Direct ubiquitin fusion to Gag can functionally compensate for the absence of a retroviral L-domain and the eventual recruitment of an ubiquitin ligase to the budding particle [10,11]. The third motif, YPX_n_L, associates with a protein named Alix (Apoptosis-linked gene 2-interacting protein) [12,13]. The 97 kDa adaptor protein provides a direct link between ESCRT-I and ESCRT-III complexes. It interacts with ESCRT-III by means of its N-terminal Bro1 domain and with Tsg101 via its C-terminal proline rich region. A central region of the protein mediates binding to the YPX_n_L motif present, for example, in the p9 protein of equine infectious anaemia virus (EIAV) [12,13]. Therefore, all three L-domain motifs appear to enter the same core scission complex, albeit by different routes.

Several retroviruses contain more than one type of L-domain motif, and these are often closely spaced or even overlapping. For example, Mason Pfizer monkey virus (MPMV), a close relative of HERV-K(HML-2), harbors a PSAP sequence four amino acids downstream of a PPPY motif within its pp24/16 protein [14]. Furthermore, the human T-cell leukemia virus type I (HTLV-I) contains a bipartite *PPPY*VE*PTAP* motif and HIV-1 has, in addition to its primary PTAP motif, an additional L-domain of the YPX_n_L type [13,15]. Beside the L-domains, the nucleocapsid protein is also engaged in the budding process through an interaction with the Bro1 domain of Alix [16].

While the budding of exogenous retroviruses has been well characterized, little is known about this process for endogenous retroviruses, although a PTAP motif was recently identified in the Gag of the human endogenous retrovirus (HERV)-K(HML-2) [17-19]. HERVs are relicts of infectious exogenous retroviruses whose proviruses became integrated into the human genome millions or at least hundred thousands of years ago [20,21]. They comprise approximately 8% of human DNA and several of these elements have been linked to oncogenesis, neurological disorders and autoimmune diseases [22-25]. Despite the fact that all the known elements carry mutations that prevent productive replication, functional proteins and mature virus-like particles of the HERV-K(HML-2) subfamily are expressed [26]. The study of virus assembly and other aspects of these archaic retroviruses have been facilitated by the generation of consensus sequences and reconstitution of original virus sequences that allow expression of functional proteins [17,27-29].

In this present report, we show that HERV-K(HML-2) uses two distinct types of L-domain motifs to hijack the ESCRT pathway. Mutation of a single nucleotide in the PTAP motif of the HERV-K(HML-2) Gag p15 protein leads to a characteristic L-domain phenotype, with viruses arrested at a late stage of release. Our results indicate that the PTAP motif is the core of the principal L-domain of the virus. However, at least two auxiliary YPX_n_L L-domains in p15 are present that efficiently provide alternative access to the ESCRT pathway, if the primary L-domain is restricted and Alix is overexpressed allowing an assembly of the ESCRT-III complex.

## Results

### Generation and expression of a molecular clone of HERV-K113 in which non-synonymous postinsertional mutations are reverted

**Figure 1:**
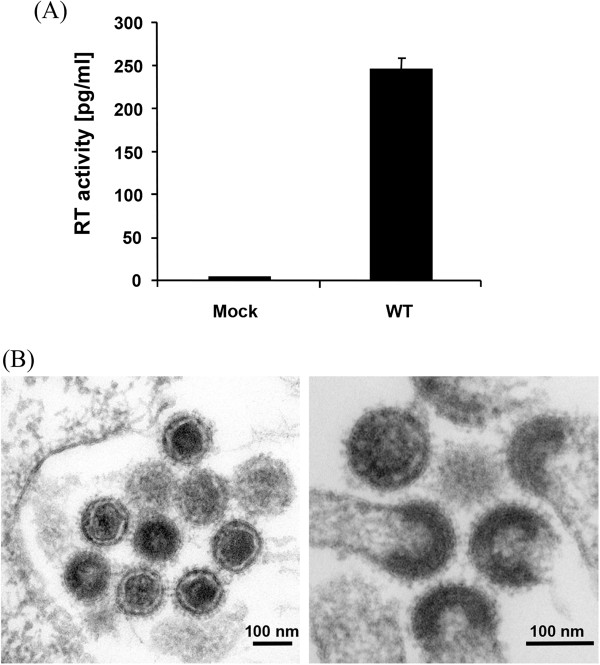
**Expression of a HERV-K113 sequence with reverted non-synonymous postinsertional mutations. (A)** Detection of RT activity in the supernatants of HEK 293T cells transfected with the molecular clone of oriHERV-K113. **(B)** Thin section EM demonstrates production of mature virions (left panel) that bud with C-type morphology (right panel).

By alignment with ten other well-preserved human-specific HERV-K(HML-2) elements, we recently identified putative non-synonymous postinsertional mutations in the *env, rec* and *gag-pro-pol* open reading frames of the HERV-K113 element described by Turner and co-workers [30] and successfully expressed the reconstituted proteins [17,28]. To generate a molecular clone of HERV-K113 expressing these reconstituted proteins (termed oriHERV-K113), the entire sequence of the element was cloned into the pBSK plasmid vector [31] and site directed mutagenesis used to revert the previously reported 25 non-synonymous putative postinsertional mutations [17,24,28]. Three additional changes (T8588C, A8799G and T9133C) were introduced in the 3′LTR to match it with the 5′LTR sequence (see Additional file 1). Silent mutations and presumed further postinsertional mutations in non-coding regions were not changed. Transfection of the pBSKoriHERV-K113 plasmid into HEK 293T cells resulted in the production and release of virus like particles as demonstrated by reverse transcriptase activity in the supernatant (Figure 1A) and thin-section electron microscopy of the producing cells (Figure 1B). The released particles were not able to establish a productive infection and replicate in HEK 293T, Tera-1, SK-Mel13 or other cell lines we have tested so far (data not shown).

### In silico screen for putative L-domains in the Gag precursor protein of the reconstituted HERV-K113 element

**Figure 2:**
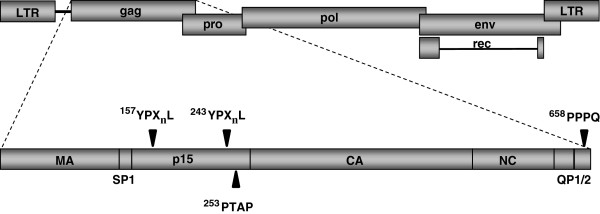
**Schematic representation of a HERV-K(HML-2) provirus and the position of presumed L-domains within the Gag protein.** Genomic structure of the provirus. The Gag protein encodes the matrix protein (MA), a spacer peptide (SP1), p15, capsid (CA), nucleocapsid (NC) and two small peptides (QP1 and QP2). A PTAP and two YPX_n_L motifs with presumed L-domain function are present in the p15 protein. A PPPQ sequence resembling a PPPY L-domain is present in QP2. The position of the first amino acid of each motif in the Gag protein of oriHERV-K113 is given.

To identify potential late domains of HERV-K(HML-2) the amino acid sequence of the oriHERV-K113 Gag precursor was screened for motifs that match or at least resemble one of the canonical L-domain sequences. During this search and evaluation we took into account the fact that retroviral late domains are frequently found in Gag regions encoding phosphoproteins located between the matrix and capsid (as for example in MPMV) and in short proteins adjacent to the nucleocapsid (as in p6 of HIV or p9 of EIAV) [32]. An exactly matching PTAP sequence was identified in the C-terminal region of p15 together with two potential YPX_n_L motifs, of which one is situated just 10 amino acids further towards the N-terminal (Figure 2). In addition, the proline-rich QP1 and QP2 peptides at the C-terminus of the Gag precursor contain PPPQ motifs that resemble the canonical PPPY sequence of L-domains directly interacting with Nedd4-like ubiquitin ligases [7,17].

### The proline-rich peptides QP1 and QP2 do not harbor late domain activity

**Figure 3:**
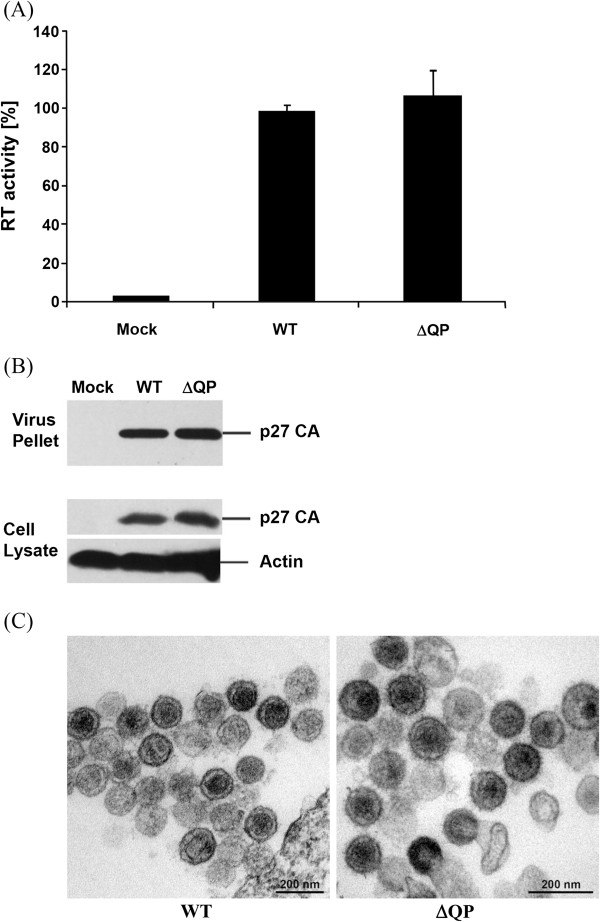
**Deletion of the QP1 and QP2 peptides do not affect HERV-K(HML-2) release. (A)** Reverse transcriptase (RT) activity in the supernatants of cells expressing the wild type oriHERV-K113 and the QP1/2 deletion mutant (ΔQP). The RT activity was normalized by cotransfection of a luciferase expression plasmid. Data represent means of four samples with error bars (standard error) of a representative experiment. The activity with the wild type virus was set at 100%. **(B)** Released viral particles were pelleted by ultracentrifugation and subjected to SDS-PAGE. The Western blot was probed with a CA-specific polyclonal serum or an actin specific antibody. **(C)** Electron microscopic analysis of the WT and ΔQP mutant morphology. Electron micrographs show mature particles released from HEK 293T cells. No differences in the morphology of the particles were detectable.

We first examined whether the 23-amino-acid-long QP1 and the 19-amino-acid-long QP2 peptides that contain a PPPQ motif play a role in HERV-K(HML-2) budding and release. The first codon of QP1 was substituted for a stop codon to terminate the Gag precursor at this site and prevent expression of both peptides. HEK 293T cells were then transfected with the wild type plasmid expressing oriHERV-K113 and the QP1/2 deletion mutant (ΔQP) along with a luciferase vector to normalize for transfection efficiency. Comparable levels of RT activity (Figure 3A) and p27 capsid protein (Figure 3B) were measured in the supernatants of the wild type and mutant. Furthermore, no evidence for a late domain phenotype was evident in thin section electron microscopy (Figure 3C). These findings indicate that the two C-terminal polypeptides do not harbor a motif able to provide significant late domain functions to the virus.

### The PTAP motif in p15 is the core of a bona fide late domain

**Figure 4:**
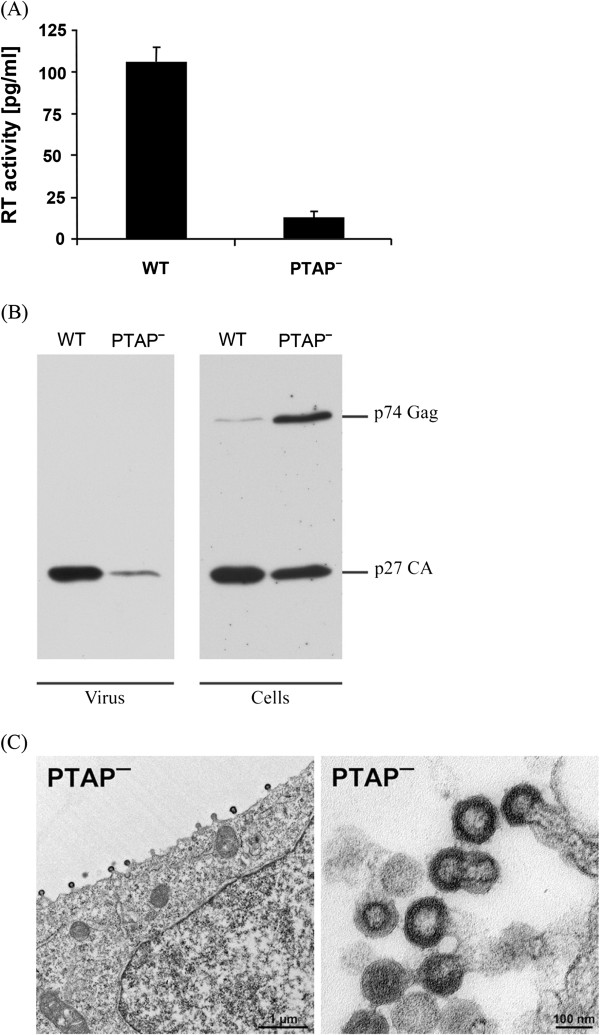
**Mutation of the PTAP motif impairs particle release and induces an L-domain phenotype. (A)** Effect of PTAP inactivation on the release of viral particles in HEK 293T cells. Data represent the mean of four experiments with standard errors. **(B)** SDS PAGE of pelleted virions (left panel) and lysates of the producer cells (right panel). The Gag precursor and processed CA proteins in cell lysates were immunoprecipitated and detected by Western blotting. The lysate of the PTAP mutant contains more p74^Gag^ precursor protein and less p27 CA. The p27 CA protein is presumably reminiscent of released mature viruses attached to the cells. **(C)** Thin section EM analysis of HEK 293T cells transfected with PTAP mutant oriHERV-K113.

Of all potential L-domains in the HERV-K(HML-2) Gag protein, the PTAP motif at position 253 is considered to be most likely a late domain because of its location and perfect match to the consensus sequence. To determine the effect of its inactivation, the threonine at position 254 was substituted with alanine (T254A) in oriHERV-K113 and the mutant designated as PTAP^-^. Supernatants from HEK 293T cells transfected with the PTAP mutant showed a 6-fold decrease in RT activity compared to wild type (Figure 4A). The reduced release of the PTAP mutant was also seen in the intensity of the p27 CA protein band in Western blots of the virus pellet (Figure 4B left panel). The Western blot analysis of the cell lysate reveals somewhat more immature Pr^74^Gag precursor and slightly less p27 CA compared to the wild type virus (Figure 4B right panel), which is in accordance with a reduced ratio of released viruses. Thin section electron microscopy of the PTAP mutant was used to visualise the potential late domain phenotype. Indeed, in contrast to the wild type (Figures 1 and 3), the PTAP mutant showed predominantly immature virus particles arrested at a late budding stage at the cell membrane (Figure 4C left panel). Furthermore, high numbers of aberrant budding structures were detected, mainly in the form of chain buds in which different procapsids are connected by membrane stalks (Figure 4C right panel).

### Tsg101 is recruited to the PTAP motif in the Gag p15 protein

Tsg101 is an essential component of the ESCRT-I complex and has been shown to interact directly with the PTAP motifs of several retroviruses [6,33,34]. We therefore investigated the subcellular colocalization of HERV-K(HML-2) Gag and Tsg101 to provide further evidence for a conventional role of this virus’s p15 protein PTAP motif. A codon-optimized version of the oriHERV-K113 *gag* sequence [17] was cloned in-frame upstream of the Cherry fluorescent protein and a PTAP mutant generated by substituting the threonine for alanine.

**Figure 5:**
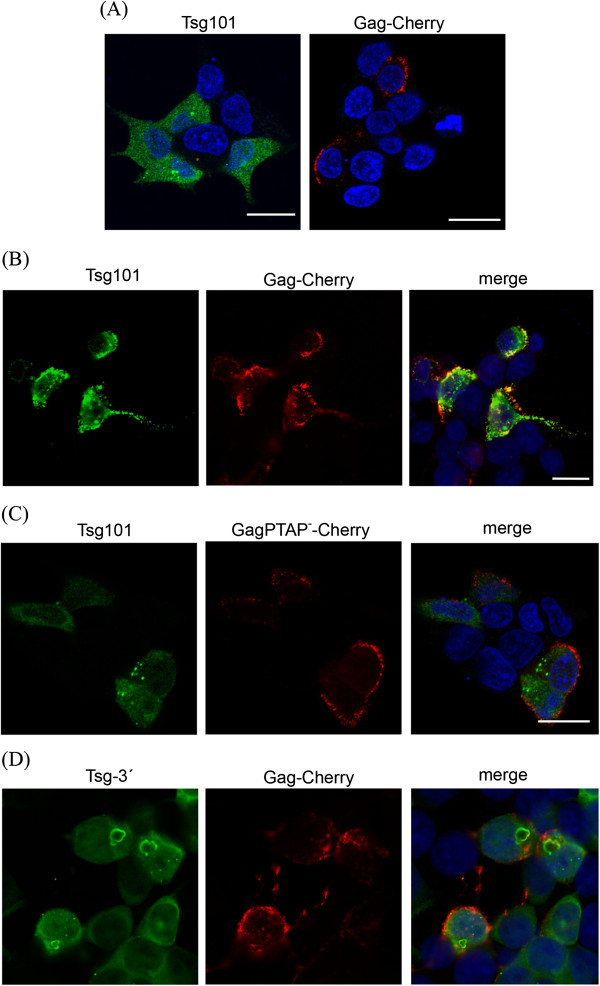
**Tsg101 is recruited by the PTAP motif of HERV-K(HML-2) Gag to sites of particle egress.** Cells were visualized by confocal microscopy as described in the material and methods. Scale bar is 20 μm. **(A)** HEK 293T cells were transfected either with a full-length HA-Tsg101 expression vector or with Gag-Cherry alone. **(B)** Colocalization of HA-Tsg101 and Gag-Cherry in cells expressing both proteins. **(C)** No colocalization is observable if GagPTAP^-^-Cherry is expressed. **(D)** HA-Tsg-3′, a mutant lacking the UEV domain, also fails to colocalize with Gag-Cherry.

Expression of both the wild type and PTAP mutant Gag-Cherry fusion proteins resulted in an accumulation of the proteins at presumed budding sites at the cell membrane (Figure 5). Coexpression of an HA-tagged Tsg101 protein revealed significant colocalization of the wild type Gag-Cherry with HA-Tsg101 (Figure 5B). Without Gag-Cherry coexpression, HA-Tsg101 expression was diffusely cytoplasmic with dot-like staining (Figure 5A), which is consistent with previous observations [35]. This indicates that Tsg101 is recruited to the Gag clusters at the cell membrane and this appears to depend on a functional PTAP motif because it was not evident during expression of the PTAP mutant (Figure 5C). Moreover, a mutant Tsg101 variant, Tsg-3′, with a deletion of the N-terminal UEV (Ubiquitin E2 variant) domain responsible for the interaction with HIV-1 p6 PTAP motif [34,36], did not colocalize with the wild type Gag-Cherry protein (Figure 5D). Tsg-3′ localization differs from full-length Tsg101 by the formation of large vacuolar structures that are not colocalized with Gag-Cherry. These results strongly suggest that the PTAP motif in the p15 protein provides the link to the ESCRT-I complex by binding to the Tsg101 protein.

### The two YPX_n_L motifs in the Gag p15 protein serve as supplementary late domains

In addition to the PTAP motif, the HERV-K(HML-2) p15 protein contains two putative, uncharacterized L-domains of the YPX_n_L type (Figure 2). To evaluate the functionality of each with regard to virus release, mutations previously shown to inactivate this type of L-domain [11,37] were introduced. In addition, double mutants with inactivated PTAP motifs and a triple mutant with inactivation of both YPX_n_L motifs and the PTAP motif were prepared (Table 1).

**Table 1:** Overview of the p15 L-domain mutants used in the study

**Mutant**	**YPX**_**n**_**L1**	**YPX**_**n**_**L2 PTAP**
WT	VI**YPETL**KLEGK	RAP**YPQPPTRRL**N**PTAP**PSR
PTAP^-^	VI**YPETL**KLEGK	RAP**YPQPPTRRL**N**P *A *** **AP**PSR
YPX_n_L2^-^	VI**YPETL**KLEGK	RAP ***SR*** **QPPTRR** ***A*** N**PTAP**PSR
YPX_n_L2-PTAP^-^	VI**YPETL**KLEGK	RAP ***SR*** **QPPTRR** ***A*** N**P *A *** **AP**PSR
YPX_n_L1^-^	VI ***SR*** **ET *A *** **K** ***A*** EGK	RAP**YPQPPTRRL**N**PTAP**PSR
YPX_n_L1^-^-PTAP^-^	VI ***SR*** **ET *A *** **K** ***A*** EGK	RAP**YPQPPTRRL**N**P *A *** **AP**PSR
YPX_n_L1^-^-YPX_n_L2^-^	VI ***SR*** **ET *A *** **K** ***A*** EGK	RAP ***SR*** **QPPTRR** ***A*** N**PTAP**PSR
YPX_n_L1^-^-YPX_n_L2^-^-PTAP^-^	VI ***SR*** **ET *A *** **K** ***A*** EGK	RAP ***SR*** **QPPTRR** ***A*** N**P *A *** **AP**PSR

**Figure 6:**
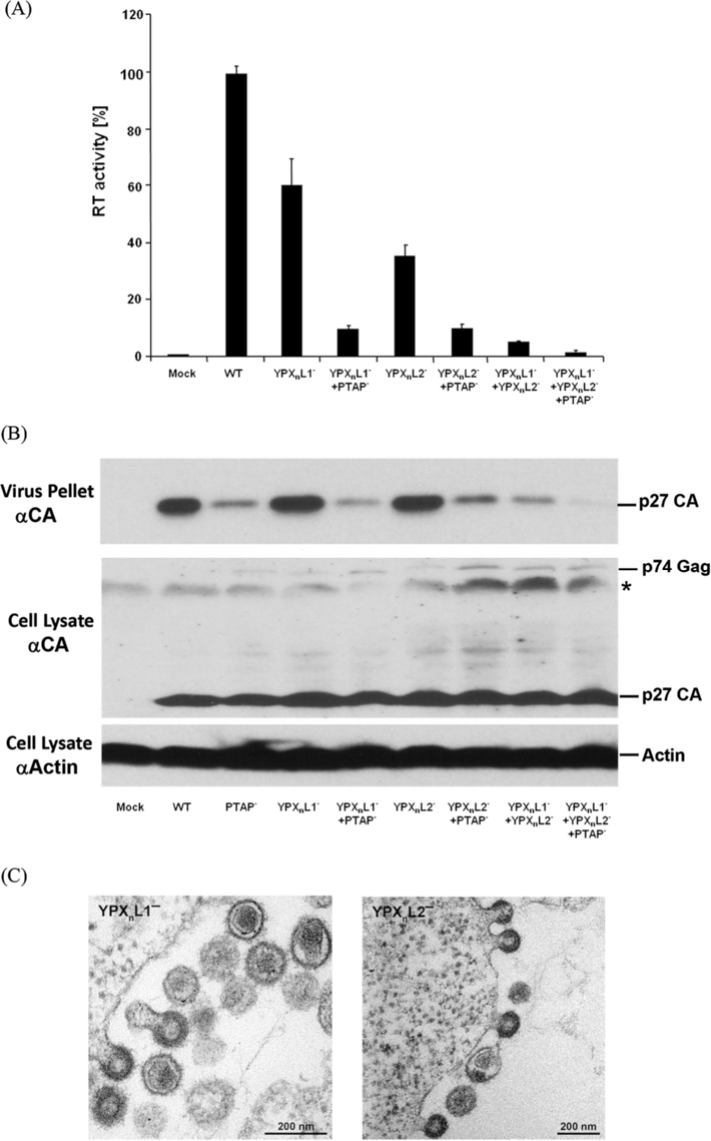
**The YPX**_**n**_**L1 and the YPX**_**n**_**L2 motifs function as additional L-domains. (A)** Comparison of the release of various L-domain mutants from transfected HEK 293T cells with that of the wild type oriHERV-K113. Means and standard errors of a representative experiment performed in quadruplicates are shown. **(B)** SDS-PAGE of virus pellets from the supernatants of HEK 293T cells transfected with WT and L-domain mutants. The Western blots were probed with a rat-anti-p27 CA serum. The cell lysates were in addition hybridized with an anti-actin antibody. A representative experiment of four performed is shown. The star depicts an unspecific band. **(C)** EM analysis of YPX_n_L defective phenotypes in HEK 293T cells.

HEK 293T cells were then transfected with the mutants and the wild type oriHERV-K113. As shown in Figure 6A, inactivation of the N-terminal YPX_n_L motif (YPX_n_L1) resulted in a 40% reduction in virus release whereas mutation of the YPX_n_L motif (YPX_n_L2) adjacent to PTAP led to a 64% decrease in supernatant RT activity. Furthermore, the impact of the double YPX_n_L mutations was significantly higher, reducing release by about 95%. Mutations of each of the single motifs resulted in a modest additional drop in the release of the PTAP mutant whereas the triple mutation reduced release to background levels (Figure 6A). These results were confirmed by Western blotting of virus pellets in which the synergistic effect of the YPX_n_L double mutant is clearly visible (Figure 6B). Moreover, using thin section electron microscopy, the typical phenotype of L-domain mutants was frequently seen in cells transfected with the YPX_n_L mutants (Figure 6C).

### Inhibition of Tsg 101 or Alix expression by RNA interference significantly reduces HERV-K(HML-2) release

**Figure 7:**
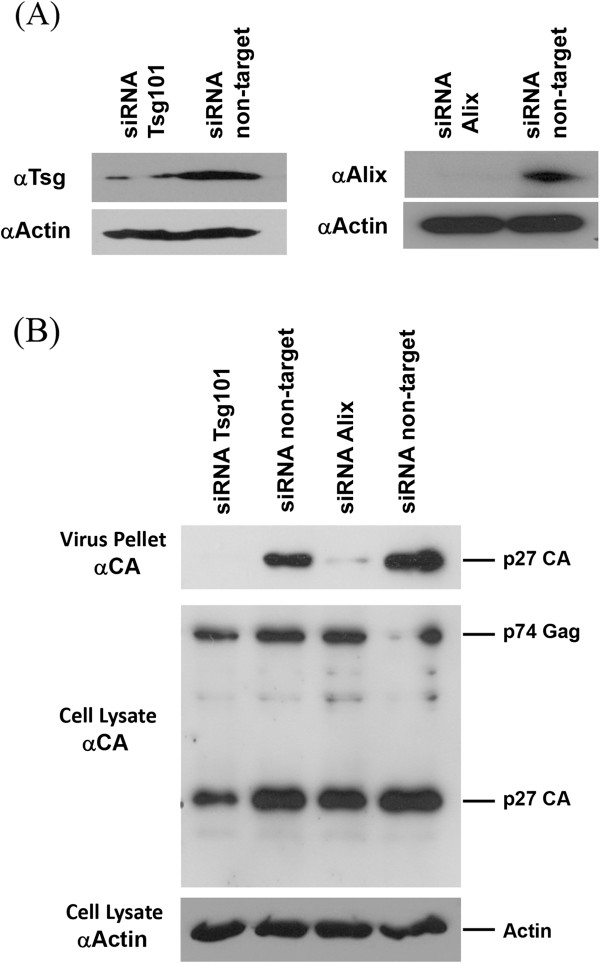
**Inhibition of Tsg101 and Alix expression by RNA interference effects HERV-K(HML-2) egress. (A)** A significant down regulation of Tsg101 (left panel) and Alix (right panel) was achieved in HEK 293T cells with siRNAs specific for the respective mRNAs. **(B)** For the determination of the impact of Tsg101 or Alix inhibition of HERV-K113 release, pBSK-oriHERV-K133 was contransfected with specific siRNAs or non-target siRNAs as control. Twenty-four hours post transfection the virus from the supernatants and the cell lysates were loaded on a Western blot. The blot was probed with a rat anti-p27 serum.

In order to corroborate an involvement of Tsg101 and Alix in the release process of HERV-K(HML-2) virions we have transfected HEK 293T cells with the pBSKoriHERV-K113 plasmid along with a mix of two siRNAs previously shown to efficiently down regulate Tsg101 or with another mix of two siRNAs targeting the Alix mRNA [6,38-40]. As expected, a significant down regulation of Tsg101 and Alix has been achieved by the specific siRNAs in contrast to a non-target siRNA used as control (Figure 7A). The inhibition of Tsg101 expression as well as the inhibition of Alix expression had both a strong effect on virus release monitored 48 hours post transfection by Western blot (Figure 7B). These results are in line with our previous findings pointing towards a major role of Tsg101 and Alix in the budding and release process of the investigated endogenous betaretrovirus.

### Overexpression of Tsg101 rescues budding of the HERV-K(HML-2) YPX_n_L mutants and Alix overexpression rescues release of PTAP mutants

**Figure 8:**
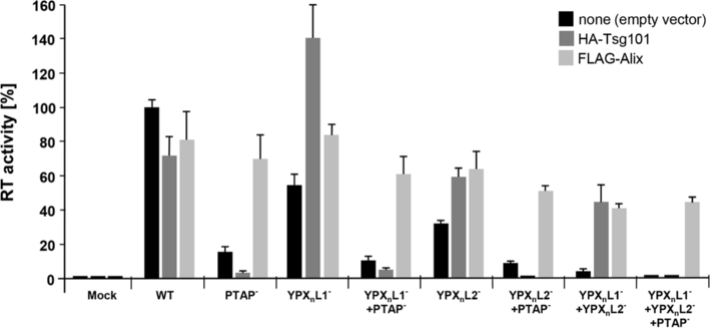
**Overexpression of Tsg101 and Alix rescue HERV-K(HML-2) defective L-domain mutants.** HEK 293T cells were transfected with WT or oriHERV-K113 L-domain mutants with and without cotransfection of HA-Tsg101 or FLAG-Alix. A luciferase vector was added for normalization. RT activities in the supernatants were determined at 48 h post transfection and normalized. The adjusted RT activities are shown in relation to the WT. The means of three experiments performed in quadruplicate are shown with standard error bars. The quantity of DNA per transfection was held constant by addition of an empty pcDNA3.1 vector.

Since full-length Tsg101 only colocalized with an intact HERV-K(HML-2) Gag PTAP domain, it was important to know whether overexpression of the protein could rescue the budding and release rates of YPX_n_L mutants. We therefore cotransfected HEK 293T cells with plasmids encoding HA-Tsg101 and with plasmids encoding either oriHERV-K113 or the various L-domain mutants. The efficiency of egress was determined by measuring RT activity in the supernatants at 48 h post transfection. Overexpression of Tsg101 was found to slightly reduce the release of wild type virus (Figure 8), as reported previously and attributed to intracellular perturbations of the endosomal compartment by overexpression of the protein [41]. In the presence of a functional PTAP motif, overexpression of Tsg101 resulted in a significant increase in the release of the YPX_n_L single and double mutants. As expected, this increase was not seen with mutants carrying a mutation in the PTAP motif. These data indicate that overexpression of Tsg101 can largely compensate for a lack of a functional YPX_n_L type L-domain and confirm the contribution of Tsg101 to the budding of HERV-K(HML-2). The data also indicate that the PTAP motif in p15 is the only site in the viral proteins that enables interaction with Tsg101. The expression of HA-Tsg101 and FLAG-Alix was confirmed by immunofluorescence (data not shown).

Analogous to the rescue of HERV-K(HML-2) YPX_n_L defects by ectopic Tsg101 expression described above, previous studies have documented that overexpression of Alix can rescue budding defects of HIV-1 PTAP L-domain mutants [12,42]. We therefore used our HERV-K(HML-2) PTAP mutants to determine whether overexpression of Alix also increases their release. Cotransfection of FLAG-Alix plasmids indeed partially abrogated the PTAP defects in mutants with one or two wild type YPX_n_L motifs. Surprisingly, release of even the virus with mutations in both YPX_n_L motifs was rescued, reaching levels of up to 50% of the wild type (Figure 8). As well as being in line with the identified functional YPX_n_L motifs, these findings also potentially indicate the presence of at least one additional, unidentified cryptic YPX_n_L motif. Alternatively, low expression and incorporation of chromosomally encoded Gag proteins into nascent viral particles might also explain the rescue by high cytoplasmic levels of Alix. Indeed, we previously demonstrated very low levels of HERV-K(HML-2) transcripts in HEK 293T cells by sensitive RT-PCR [31].

### Effect of L-domain mutants in Tera-1 cells expressing high levels of chromosomally encoded HERV-K(HML-2)

**Figure 9:**
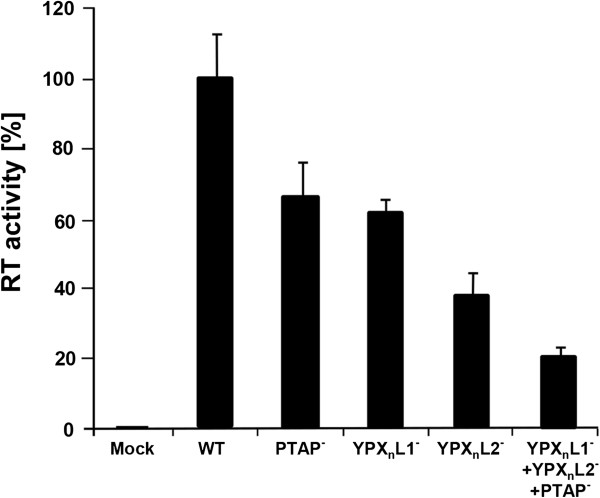
**Release of HERV-K(HML-2) L-domain mutants in Tera-1 cells.** Tera-1 cells expressing considerable amounts of HERV-K(HML-2) Gag were transfected with oriHERV-K113 or with the L-domain mutants indicated. RT activity in the supernatants was detected 48 h post transfection and the data represent means and standard errors of an experiment performed in quadruplicate. The activity obtained with the wild type was set at 100%.

To further test the hypothesis that incorporation of chromosomally encoded HERV-K(HML-2) Gag-proteins can rescue ectopically expressed HERV-K(HML-2) mutants, Tera-1 cells were transfected with plasmids encoding wild type and mutants of oriHERV-K113. Tera-1 cells are known to express HERV-K(HML-2) Gag proteins, including those with preserved L-domains [43]. Indeed, especially the release of the PTAP mutant was significantly less impaired compared to HEK 293T cells. In contrast to the virtually complete block in HEK 293T cells, the triple mutant showed only an 80% reduction in particle release (Figure 9). These results support the possibility that chromosomally encoded Gag proteins are incorporated into nascent particles, rescuing release of ectopically expressed L-domain mutants.

## Discussion

The human genome encodes about 90–100 well-preserved proviruses of the HERV-K(HML-2) family [44]. Several of these proviruses code for Gag proteins that can form retroviral particles upon expression. Using electron microscopy and other methods, we and others have demonstrated that these particles bud in a C-type manner and are in principle able to ‘pinch off’ from the cell membrane and undergo maturation [17,27,45]. To identify the protein motifs of HERV-K(HML-2) that govern the release of these ancient retroviruses, we generated a reconstituted HERV-K113 element in which non-synonymous postinsertional mutations were reverted (termed oriHERV-K113). The lack of such mutations renders consensus sequences or reconstituted sequences of endogenous retroviruses and retroelements suitable for general functional analyses of their proteins [17,27-29,46].

Guided by knowledge of the common position and consensus sequences of late domains in exogenous retroviruses, we performed an *in silico* screen of the oriHERV-K113 sequence and identified a potential PTAP motif and two YPX_n_L motifs in the p15 protein localized between the matrix and capsid subunits of Gag. One of the YPX_n_L domains, a *YP*ET*L* sequence, is found N-terminal in the p15 protein. The second, a *YP*QPPTRR*L* sequence, is located just 10 amino acids upstream of the PTAP motif with only a single intervening amino acid. Closely spaced L-domains are very frequently observed in retroviruses and some other enveloped viruses [47,48]. In the lentivirus HIV for example, a PTAP and a YPX_n_L L-domain is located in the phosphoprotein p6 at the C-terminus of the Pr55^Gag^[3]. HERV-K(HML-2) encodes two proline and glutamine rich peptides at the C-terminus with unknown functions [17]. Although a conventional L-domain motif is not evident in these peptides, sequences closely resembling a PPPY motif are present, making this a region of interest. Deletion mutants unable to express these peptides showed no impairment or defect in particle release, strongly arguing against them having an L-domain function.

In contrast to this C-terminal deletion mutant, a T254A substitution in the PTAP sequence (known to inactivate this L-domain) reduced particle release in HEK 293T cells by a factor of approximately six, and thin section EM revealed a classical late domain phenotype with an abundance of particles at the membrane unable to finalize the last steps of release and ‘pinch off’. A similar defect in particle release in a PTAP mutant of the HERV-K(HML-2) consensus sequence has also been recently reported [18]. The function of this sequence as core of an L-domain has been further substantiated by colocalization studies of the HERV-K(HML-2) Gag with the ESCRT-I complex protein Tsg101. While full length Tsg101 is found in association with Gag at presumed budding sites, this is not the case if the PTAP mutant is expressed, indicating that the PTAP motif at position 253 is the only Tsg101 binding site for this virus’s Gag protein. This is supported by the fact that overexpression of Tsg101 fails to increase the release of the PTAP mutant. Consistent with previously published observations with HIV and other viruses, overexpression of Tsg101 resulted in a moderate decrease of wild type release that can be explained by a partial disorder of cellular endosomal sorting pathways [41,49]. On the other hand, transfection of HEK 293T cells with a Tsg101 expression plasmid increased significantly the release of both YPX_n_L L-domain mutants and of the version carrying mutations in both YPX_n_L motifs. Inactivation of each of the presumed two YPX_n_L L-domains reduced particle release and induced a late phenotype, although less dramatically than did mutation of the PTAP motif. By thin section EM we observed changes in quantity but no morphologic differences in the PTAP and the YPX_n_L mutants.

The impact of the mutations regarding the degree of virus release indicates that in HEK 293T cells the PTAP motif is the dominant L-domain and each of the YPX_n_L motifs play a supportive role. We obtained similar results with SK-Mel13 cells, with a more pronounced inhibition (up to 95%) of release by the PTAP mutant (data not shown). However, in other cell types the PTAP motif might be of less relevance if, for example, more Alix and less Tsg101 is expressed. The relevance of different types of L-domains is known to be cell type dependent [50]. For efficient budding retroviruses must recruit the ESCRT-III complex. It provides the mechanical means for scission of the virus from the cell membrane. This complex can be recruited via the ESCRT-I or ESCRT-II route, though Alix appears to be used in both pathways. Alix interacts with the ESCRT-III complex so that by high Alix levels or overexpression of this protein the downstream ESCRT-III complex is assembled and budding can be restored. However, the process is much more efficient if the ESCRT-I complex is available [51].

In HEK 293T cells, mutation of both YPX_n_L L-domains has a synergistic effect on particle release compared with that of individual motif mutants, resulting in a stronger inhibition than that seen with the PTAP mutation. This is in line with the significant reduction in particle release following siRNA mediated down regulation of Alix or Tsg101 expression. The close proximity of the PTAP motif to one of the YPX_n_L L-domains suggests that mutation in one of these domains might also inactivate the other. This, however, appears not to be the case as variants carrying mutations in both motifs (YPX_n_L2 + PTAP) are more impaired than the single motif mutants. Moreover, overexpression of Tsg101 is able to partially rescue the release of the variant carrying an inactivating mutation in the nearby YPX_n_L motif, demonstrating the presence of a functional PTAP motif. In turn, Alix overexpression can partially rescue a virus that carries inactive PTAP and N-terminal YPX_n_L1 motifs, providing further evidence that YPX_n_L motifs function as L-domains in HERV-K(HML-2) Gag. However, the triple mutant carrying inactivating mutations in both YPX_n_L L-domains plus the PTAP domain is still partially rescued by Alix overexpression. Therefore, there may be one or more additional cryptic sites with low affinity for Alix that allow interaction with this protein (if overexpressed) and provide access to the ESCRT machinery. Alternatively, low-level expression of Gag from proviruses encoded in the chromosomes of HEK 293T cells might, under such circumstances, be sufficient to allow release of this mutant. Although HEK 293T cells express HERV-K(HML-2) transcripts at barely detectable levels [31], incorporation of Gag from endogenous proviruses with functional L-domains is very likely since even HIV Pr55^Gag^ has been shown to be integrated into nascent HERV-K(HML-2) particles [18]. Even a minor fraction of Gag proteins with functional L-domains per particle is sufficient to confer egress from the cell [14] and our results with Tera-1 cells that express considerably higher levels of endogenous Gag support this hypothesis. The expression of a minority of Gag proteins with functional L-domains might be the reason why many tumor cells are able to release HERV-K(HML-2) particles. The particle production of the PTAP and triple mutants in Tera-1 was only moderately impaired, with a 5-fold reduction in the release of the triple mutant compared to complete inhibition in HEK 293T cells. However, further experiments are needed to confirm or refute the presence of cryptic Alix binding sites in the HERV-K(HML-2) Gag protein and to analyze the effects of incorporation of low fractions of Gag proteins with functional L-domains.

## Conclusions

This study identifies three L-domains in a partially reconstituted prototypical HERV-K(HML-2) virus. The major L-domain, providing access to the ESCRT complex via the Tsg101 protein, is a consensus PTAP motif in the C-terminus of the p15 protein. Based on mutagenesis studies and rescue experiments with Alix overexpression, this L-domain was shown to be supported by two auxiliary YPX_n_L motifs also located in the p15 protein of the virus. By providing essential information concerning the relevant functional domains facilitating HERV-K(HML-2) particle release, these results advance our understanding of the biology of these ancient and inherited elements preserved within our genomes.

## Methods

### Cell culture

HEK 293T, SK-Mel13 and Tera-1 cells were cultured in complete Dulbecco’s modified Eagle medium (DMEM) supplemented with 10% fetal bovine serum, L-glutamine (2 mM), penicillin (50 U/ml) and streptomycin (50 μg/ml).

### Plasmid DNA construction

For the generation of the pBSKoriHERV-K113, the proviral sequence of HERV-K113 (GenBank AY037928) was amplified from the RP11-12X BAC-library plasmid [30] and cloned into the small pBlueskript SK+ (pBSK) vector from Stratagene using Apa I and Not I as restriction sites. The identification of the presumed 25 non-synonymous postinsertional mutations has been described elsewhere [17,24,28]. These mutations and the three mutations in the 3′LTR (see Additional file 1) were introduced into pBSK-HERV-K113 by site-directed mutagenesis using the QuikChange® Multi Site-Directed Mutagenesis Kit from Agilent Technologies to produce pBSKoriHERV-K113.

The L-domain mutants were also generated by site-directed mutagenesis using the same Kit and the following primers: PTAP^-^: 5′-gccgcccactaggagacttaatcccgcggcaccacctagtagacagggtagtg-3′; YPX_n_L1: 5′-caattacaggaggtgatatctagagaaacgttaaaattag-3′ and gtatggatatctagagaaacggcaaaagcagaaggaaaaggtccag; YPX_n_L2: 5′-ggcagggcgccatccagacagccgcccactaggag-3′ and 5′-cagccgcccactaggagagctaatcctacggcaccac-3′. The primer 5′-caacaaactggggcattctgaattcagccatttgttcc-3′ was used for the introduction of the stop codon after NC to generate the ΔQP-mutant and the primer 5′-cctcccaccaggcggctgaacgcccctcccagcaggcagagcgag-3′ to introduce the PTAP^-^-mutation into the codon-optimized Gag in the pGag-Cherry vector. The reverse complement oligonucleotides primers used in the mutagenesis reactions are not shown.

The pGag-Cherry construct was designed for immunofluorescence microscopy in which the synthetic partially codon-optimized *gag* sequence (described previously) was cloned using the Sac I and Kpn I sites of a pmCherry-N1 vector [17,28]. The PTAP mutant pGagPTAP^-^-Cherry was generated as described above using the QuikChange® Multi Site-Directed Mutagenesis Kit (Agilent Technologies). pcGNM2/Tsg-F and pcGNM2/Tsg-3′, used to express Tsg101 and Tsg-3′, respectively, were kind gifts of Eric Freed (University of Maryland, USA). The plasmid pCMV-FLAG-ALIX was kindly provided by Jörg Votteler (University of Erlangen, Germany). The small interfering RNAs (siRNA) used to down regulate Tsg101 has been previously described [6,38] as well as the Alix specific siRNAs [39,40]. As non-target siRNA the “AllStars Neg. Control siRNA” (Qiagen) was used.

### RT activity and luciferase assays

HEK 293T cells (6 × 10^5^) were seeded into 6-well plates and transfected the following day with 1.95 μg pBSKoriHERV-K113 or mutant constructs using Polyfect (Qiagen) according to the manufacturer’s instructions. In addition, 0.05 μg pGL3-Promotor Vector (Promega) was included for normalization. Samples of cell culture media taken 48 h after transfection were filtered (0.45 μm) and RT activity measured using the HS-Mg RT Activity Kit (Cavidi, Uppsala, Sweden). Luciferase activity in transfected cells lysed 48 h post transfection (using the Luciferase Cell Culture Lysis 5x Reagent from Promega) was measured using the Promega Luciferase Assay Kit.

### Virus particle purification and preparation of cell lysates

2.4 × 10^6^ HEK 293T cells grown in 100 mm dishes were transfected with the different plasmids (25 μg each) using calcium phosphate. At four days post transfection, samples of culture media were harvested, clarified at 3345 × g for 8 min and filtered (0.45 μm) to remove cell debris. The supernatants were then centrifuged at 175,000 × g for 3 h at 4°C through a 20% sucrose cushion in a Beckman SW32Ti rotor. Virus pellets were resuspended in 60 μl 0.05 M Hepes buffer, pH 7.2 for Western blot analysis. To prepare cell lysates, transfected cells were resuspended in cell lysis buffer (1% Triton-X 100, 20 mM Tris pH 7.7, 150 mM NaCl) containing complete protease inhibitor cocktail (Roche Diagnostic). For the Tsg101 and Alix depletion assays, 4 μg of pBSKoriHERV-K113 were cotransfected with a mix of 6 μl of two specific siRNAs (20 μM) or the same amount (12 μl, 20 μM) of control siRNA using Attractene (Qiagen) according to the transfection protocol. The transfections were performed in 6-well plates in triplicates. For Western blot analyses the supernatants and the cell lysates of the triplicates were combined.

### SDS PAGE and western blot analysis

Viral lysates or pelleted virus particles were mixed with sample buffer and boiled for 10 min before being subjected to sodium dodecyl sulphate-polyacrylamide gel electrophoresis (SDS-PAGE). The proteins were transferred onto a PVDF membrane (Roth) and after blocking in blocking buffer (phosphate-buffered saline-PBS, 5% skim milk powder, 0.1% Tween), the membranes were probed with anti-CA rat sera as described previously [17] and a secondary horseradish peroxidase-conjugated goat-anti-rat antibody (Sigma-Aldrich). For the detection of Tsg101 the mouse monoclonal antibody 4A10 (Genetex) was used and for the detection of Alix a goat polyclonal IgG (Q-19; Santa Cruz Biotech) was applied as primary antibody with species specific secondary antibodies in both cases. The β-actin was detected with the AC-74 monoclonal antibody (Sigma-Aldrich). Proteins were visualized using Super Signal West Femto Maximum Sensitivity Substrate (Thermo Scientific) and a Kodak Medical X-ray film.

### Immunofluorescence microscopy

HEK 293T cells were cultured in chamber slides (Nunc) and transfected using Polyfect (Qiagen). Twenty-four hours post transfection, cells were fixed at room temperature with 2% paraformaldehyde in PBS for 30 min. Cells were then rinsed three times with PBS and permeabilized with 0.5% Triton-X100. After blocking with 1% skim milk powder in PBS for 30 min, cells were incubated with primary mouse anti-HA antibody diluted 1:200 in blocking buffer for 1 h at 37°C. Cells were then washed three times with PBS and incubated with a secondary Alexa-488 conjugated anti-mouse-IgG antibody (Invitrogen) for 1 h. After repeated washings, cell nuclei were stained using DAPI (4′,6-diamidino-2-phenylindole) at 0.2 ng/ml. Finally, the cells were mounted in Mowiol and examined using a Zeiss LSM 780 confocal laser scanning microscope.

### Electron microscopy

Transfected HEK 293T cells were fixed using 2.5% glutaraldehyde in 0.05 M Hepes (pH 7.2) for 1 h at room temperature. The methods for embedding and for the preparation of thin section transmission EM images have been described elsewhere [17].

## Competing interests

The authors declare that they have no competing interests.

## Authors’ contribution

CC performed most of the experiments and drafted the paper. NBa conceived the study and coordinated the experiments. MG generated the QP mutants and contributed experimental data. VL contributed EM data. AZ prepared the Gag-Cherry constructs and performed experiments. KH and NBe cloned and reconstituted the HERV-K113 sequence. All authors read and approved the final manuscript.

## Supplementary Material

Additional file 1 **Mutations introduced into HERV-K113 to generate oriHERV-K113.** Nucleotides are numbered according to the sequence in GenBank AY037928. Amino acid numbering of Gag, Env and Rec starts with the initiation codon of the proteins. Click here for file
