# Supplementary material for: Identification of late assembly domains of the human endogenous retrovirus-K(HML-2)
Source: Retrovirology. 2013 Nov 19;10:140. doi: 10.1186/1742-4690-10-140 (PMC3874623; doi:10.1186/1742-4690-10-140)
Supplement: Additional file 1 — Mutations introduced into HERV-K113 to generate oriHERV-K113. Nucleotides are numbered according to the sequence in GenBank AY037928. Amino acid numbering of Gag, Env and Rec starts with the initiation codon of the proteins. [file 1742-4690-10-140-S1.pdf]

Supplementary Table 1: Mutations introduced into HERV-K113 to generate oriHERV-K113. Nucleotides are numbered according to the sequence in GenBank AY037928. Amino acid numbering of Gag, Env and Rec starts with the initiation codon of the proteins.

**no.**	**gene**	**nucleotide**	**amino acid**
1	gag	C1551T	A147V
2		A1622G	M171V
3		A1773G	Q221R
4		T1974C	L288P
5		G2659C	M516I
6	pro	C3404T	T to I
7		A3613G	S to G
8		T3752C	V to A
9	pol	C4433A	D to E
10		T4741C	V to A
11		A4812G	T to A
12		G4891A	R to Q
13		T4924C	I to T
14		T5320C	L to P
15		C6214T	P to L
16		G6425A	M to I
17	env	A6823G	I125V
18		T6868C	C140R
19		C7234A	L262I
20		G7417C	A323P
21		T7968A	N506K
22		T8179C	C577R
23		A8326C	T626P
24	env / rec	A8414G	Y655C / T89A
25	rec	C8437A	D96E
26	3´LTR	T8588C	
27		A8799G	
28		T9133C	
